# Targeting Macrophage Dysregulation for Viral Infections: Novel Targets for Immunomodulators

**DOI:** 10.3389/fimmu.2021.768695

**Published:** 2021-11-01

**Authors:** Monica D. Reece, Ruby R. Taylor, Colin Song, Christina Gavegnano

**Affiliations:** ^1^ Department of Pathology and Laboratory Medicine, Emory University, Atlanta, GA, United States; ^2^ Miller School of Medicine, University of Miami, Miami, FL, United States; ^3^ Department of Chemistry, Emory University, Atlanta, GA, United States

**Keywords:** macrophage, immune dysregulation, SARS-CoV-2, immunomodulators, inflammation, HIV-1

## Abstract

A major barrier to human immunodeficiency virus (HIV-1) cure is the latent viral reservoir, which persists despite antiretroviral therapy (ART), including across the non-dividing myeloid reservoir which is found systemically in sanctuary sites across tissues and the central nervous system (CNS). Unlike activated CD4+ T cells that undergo rapid cell death during initial infection (due to rapid viral replication kinetics), viral replication kinetics are delayed in non-dividing myeloid cells, resulting in long-lived survival of infected macrophages and macrophage-like cells. Simultaneously, persistent inflammation in macrophages confers immune dysregulation that is a key driver of co-morbidities including cardiovascular disease (CVD) and neurological deficits in people living with HIV-1 (PLWH). Macrophage activation and dysregulation is also a key driver of disease progression across other viral infections including SARS-CoV-2, influenza, and chikungunya viruses, underscoring the interplay between macrophages and disease progression, pathogenesis, and comorbidity in the viral infection setting. This review discusses the role of macrophages in persistence and pathogenesis of HIV-1 and related comorbidities, SARS-CoV-2 and other viruses. A special focus is given to novel immunomodulatory targets for key events driving myeloid cell dysregulation and reservoir maintenance across a diverse array of viral infections.

## Introduction

**Figure 1:**
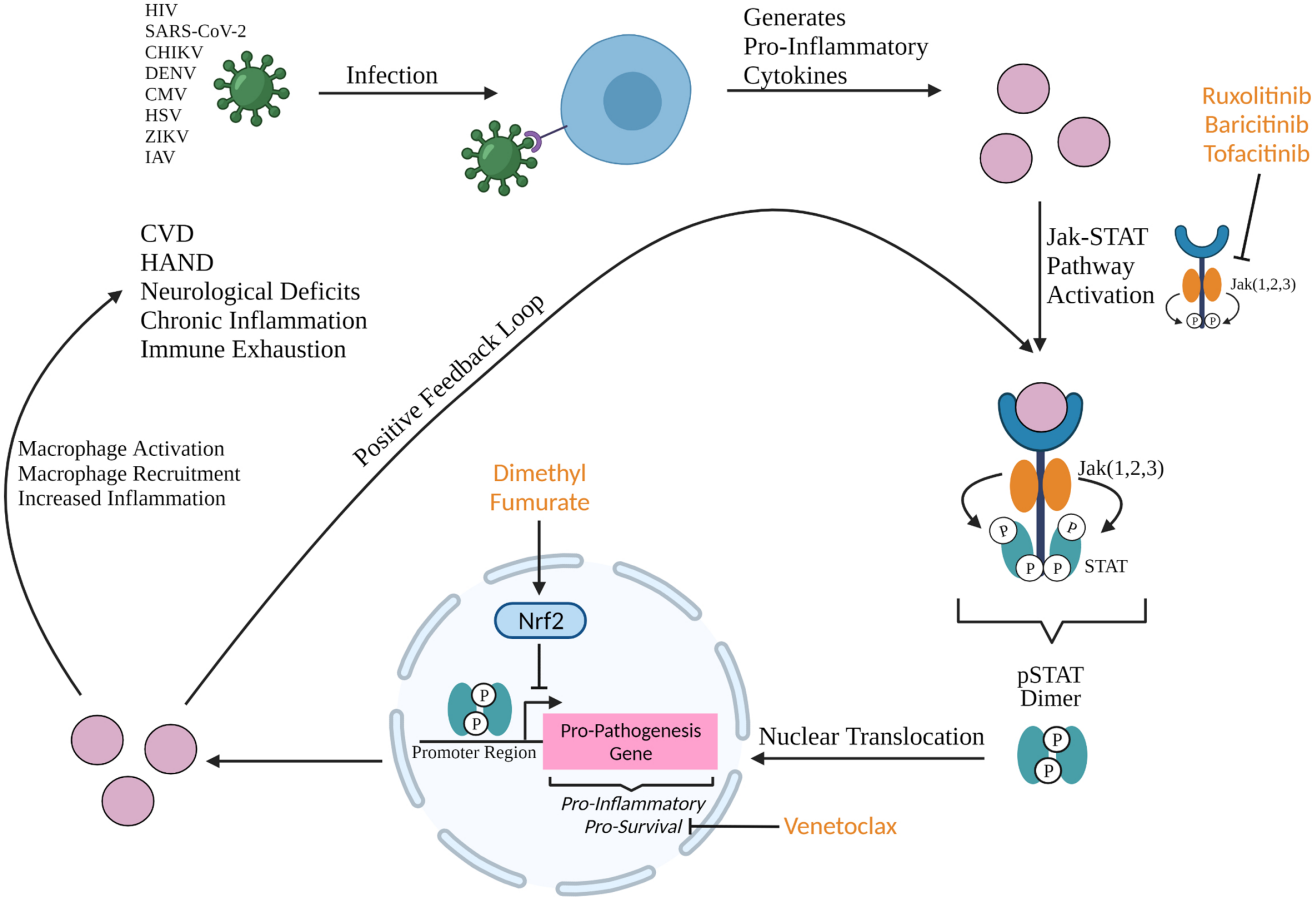
Production of pro-inflammatory cytokines upon viral infection can activate the Jak STAT pathway resulting in the formation of a phosphorylated STAT (pSTAT) dimer that binds to the promoter region of pro-inflammatory and pro-survival genes. Upregulation of these genes result in macrophage activation and recruitment, and increased systemic inflammation which all contribute to the development of neurological deficits, chronic inflammation, immune exhaustion, and comorbidities such as HIV-1-associated neurocognitive disorder (HAND) and cardiovascular disease (CVD). Jak ½ selective inhibitors ruxolitinib and baricitinib, and Jak 3 selective inhibitor tofacitinib are FDA approved compounds that are candidates to be repurposed in the anti-viral space due to their efficacy in blocking the inflammatory Jak STAT cascade. Baricitinib has been approved (EUA) for the indication of hospitalized COVID-19 patients and tofacitinib has shown significant benefit in treatment of COVID-19 (3–5). Dimethyl fumarate and venetoclax, both FDA approved compounds, are candidates to be repurposed at the transcriptional and post-transcriptional levels. Dimethyl fumarate is an activator of Nrf2 which inhibits the promotor of pro-inflammatory genes during transcription. Venetoclax is a BCL-2 inhibitor that post-transcriptionally sequesters pro-survival protein BCL-2. Created with BioRender.com.

In a regulated immune system, macrophages are phagocytic cells that target and break down foreign bodies and regulate lymphocyte activation and recruitment. M1 subtype macrophages are pro-inflammatory, differentiated through GM-CSF, TNF-α, and IFN-α cytokines, while M2 subtype macrophages are anti-inflammatory macrophages, differentiated through IL-4, IL-13, and IL-10 (1, 2). Macrophage dysregulation results in increased production of pro-inflammatory cytokines leading to accumulation of M1 macrophages and activated immune cells and increased systemic inflammation. Macrophage dysregulation as a result of disease, communicable or otherwise, causes persistent inflammation that leads to comorbidity development. Macrophage dysregulation in viral pathogenesis is an exploitable space for drug development and should be focused on because it presents a broad target as a pathogenic feature shared by multiple viruses (**Figure 1**).

## Macrophage Dysregulation in Viral Pathogenesis

**Figure 2:**
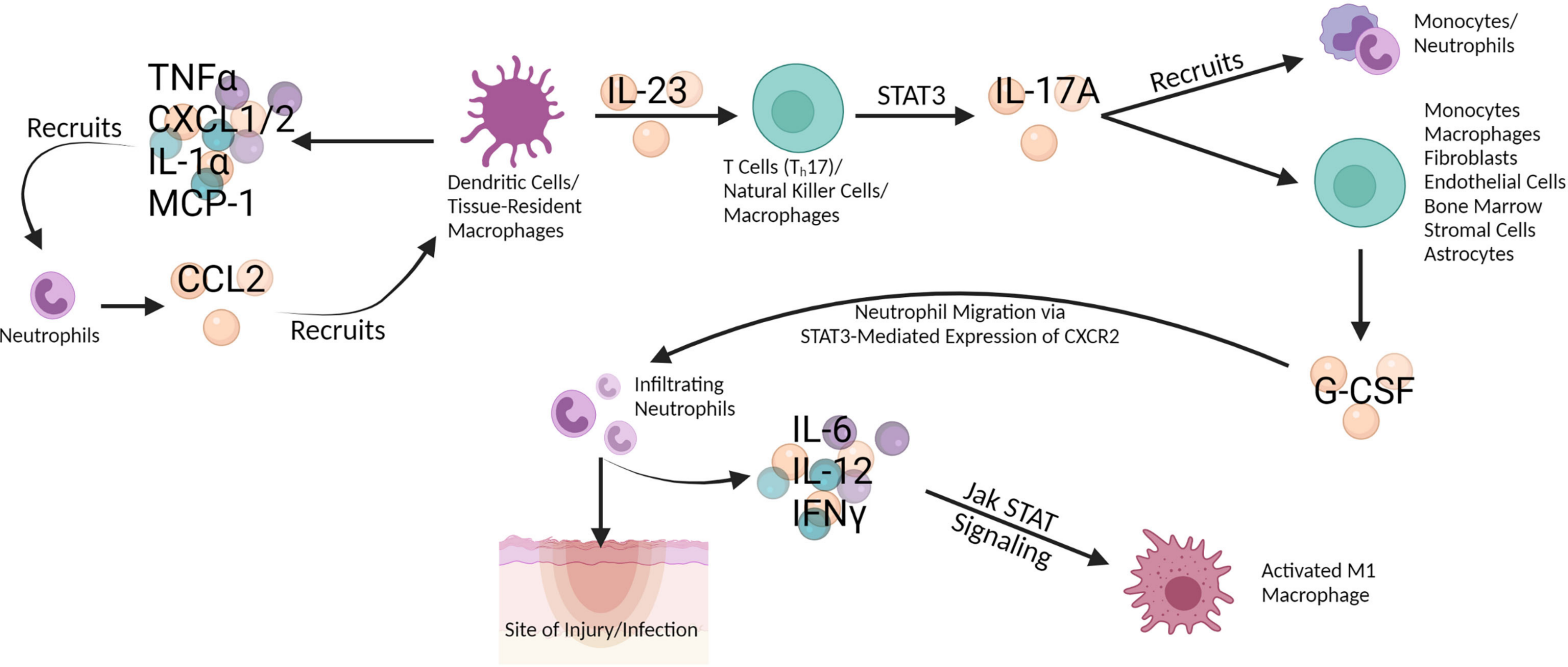
Dendritic cells (DC) and tissue-resident macrophages secrete TNF-α, CXCL1/2, IL-1 α, and MCP-1 which recruit neutrophils. In turn, the neutrophils secrete CCL2 which recruits DC and macrophages, effecting a positive feedback loop. DC and macrophages also secrete IL-23 which stimulates T cells like Th17, natural killer (NK) cells, and macrophages to produce IL-17A *via* STAT3 signaling in the Jak STAT pathway. IL-17A both recruits monocytes and neutrophils and induces G-CSF production in multiple cells types (monocytes, macrophages, fibroblasts, endothelial cells, bone marrow, stromal cells, and astrocytes). G-CSF promotes neutrophil migration to the site of injury or infection *via* STAT3-mediated expression of CXCR2. Infiltrating neutrophils secrete IL-6, IL-12, and IFN-γ, which activate pro-inflammatory (M1) macrophages through Jak STAT signaling. Created with BioRender.com.

Neutrophils interact with macrophages and other immune cell subsets at the beginning of the inflammatory cascade discussed here, often during the innate immune response (**Figure 2**). Neutrophils, myeloid leukocytes, are the first of the white blood cells to enter traumatized and diseased tissues where the pathogen is residing (6, 7). Neutrophils are constantly generated in the bone marrow and mature *via* granulocyte colony stimulating factor (G-CSF) (6). G-CSF promotes neutrophil activation, differentiation, and migration through Jak STAT signaling involving STAT3 (8–10). Endothelium activation releases several pro-inflammatory cytokines (IL-1β, IL-6, and IL-8) which results in neutrophil margination (7). Neutrophils are mediators of the acute inflammatory response (11). After microbial challenge, tissue-resident macrophages produce cytokines (TNFα, CXCL1/2, IL-1α, and MCP-1) which recruit neutrophils to the site of infection. These neutrophils then produce azurocidin resulting in the upregulation of E-selectin and vascular cell adhesion molecule-1 (VCAM-1) expression on the endothelium which promotes monocyte recruitment. Infiltrating neutrophils also secrete cytokines (IL-6, IL-12, and IFNγ) that contribute to activation of pro-inflammatory macrophages and T helper cell differentiation through Jak STAT signaling (10, 12). GM-CSF production by pro-inflammatory macrophages elongates neutrophil lifespan and induces monocyte differentiation. Following inflammation resolution, pro-inflammatory macrophages bind to neutrophils *via* TNF, induce apoptosis, and clear the apoptotic neutrophils (6, 13). A study looking at neutrophils in systemic inflammation response syndrome (SIRS) in a murine model found that mild SIRS induced a pro-inflammatory phenotype (IL-12+CCL3+) while severe SIRS induced an anti-inflammatory phenotype (IL-10+CCL2+) (6, 14). G-CSF production (monocytes, macrophages, fibroblasts, endothelial cells, and bone marrow stromal cells) is regulated by IL-17A which is produced by T cells, natural killer (NK) cells, and macrophages (6, 11, 15). Neutrophils produce CCL2 and CCL20 which are critical for recruitment of T helper cells, a major producer of IL-17A (10, 16). IL-17A production is regulated by IL-23, produced by tissue-resident macrophages and dendritic cells (DC) (6). IL-17A is produced through STAT3 activation and recruits monocytes and neutrophils, as well as stimulates astrocytes to produce G-CSF and CXCL1 in the CNS; this latter mechanism can promote astrogliosis and CNS inflammation, contributing to CNS immune dysfunction and eventual associated cognitive deficits (10, 11, 17). It is clear that neutrophil interaction with macrophages and other immune types in the beginning of the inflammatory cascade is critical and that Jak STAT signaling, and the blockade thereof, could have effects even earlier than the stage at which macrophage function becomes dysregulated.

### Human Immunodeficiency Virus

Human Immunodeficiency Virus 1 (HIV-1) is a persistent public health problem with an estimated 37.6 million people with HIV-1 (PWH) globally as of 2020 (18). The main barrier to HIV-1 cure is the viral reservoir which is comprised of transcriptionally limited/silent infected cells that are poorly affected by current antiretroviral therapy (ART) due to penetration capabilities across the blood-brain barrier (BBB) and tissues and differences in effective doses between cell types. ART has been shown to be ineffective in eradicating latently infected cells and blocking reservoir establishment, and there is no approved therapy that directly targets the reservoir (19, 20).

In recent years, the HIV-1 myeloid reservoir has become accepted as a principal reservoir that contributes to viral persistence at the hands of several studies that have shown that macrophages are directly infected, are long-lived, and are a productive source of replication competent virus. An early view of macrophage ontogeny was that macrophages are terminally differentiated and replenished *via* bone marrow derived monocytes but was expanded by new evidence of long-lived tissue resident macrophages that were derived from yolk-sac progenitors and fetal liver derived monocytes (21–24). This long-lived self-renewing phenotype placed macrophages in a new light for their potential as reservoir cells beyond their known contribution to bystander effects. When CD4+ T cells are depleted *in vivo*, viral replication is sustained by infected tissue resident macrophages, underscoring their importance as a viral reservoir (25, 26). Sterile alpha motif and HD domain-containing protein 1 (SAMHD1) is a host factor dNTPase that exists in high levels in non-dividing cells such as macrophages and reduce dNTP availability. In HIV-1 infected macrophages, reduced dNTP availability during reverse transcription results in restricted viral replication leading to infected survivor cells that produce low levels of virus. In mitotically active cells, SAMHD1 exists at lower levels resulting in more available dNTPs and thus unrestricted viral replication leading to cell death (27–30). SAMHD1 is a major factor in maintenance of the myeloid reservoir and contributes to the long-lived phenotype of infected cells.

The myeloid HIV-1 reservoir exists in the periphery, tissues, and central nervous system (CNS). Macrophage migration into tissues facilitates spread of infection to the brain, gut, lung, lymph nodes, liver, semen, and urethra (25). HIV-1 can traverse the BBB and establish infection in the CNS which is a sanctuary site because of poor penetration of ART (25). Replication competent virus has been isolated from human urethral macrophages and lymphoid tissues in macaques (22, 25, 31–33). Tissue resident macrophages were found to be a highly productive source of HIV-1 during opportunistic co-infection in lymph nodes (34).

HIV-1 infection and subsequent viremia and gut microbial translocation triggers acute inflammation. Resulting pro-inflammatory cytokines recruit macrophages and other innate immune cells, compounding macrophage activation and infection, crossing the line into macrophage dysregulation, and leading to immune exhaustion and persistent inflammation in the host (25).

### Severe Acute Respiratory Syndrome Coronavirus 2

Excessive myeloid cell and cytokine storm activation is associated with disease severity in coronavirus disease (COVID-19). Monocytes from infected individuals express angiotensin converting enzyme 2 (ACE2), the receptor for SARS-CoV-2, which suggests that monocytes are infected. Monocyte infection results in immune activation, inflammatory response, and altered gene expression related to immune signaling. M1 macrophages accumulate in the lungs and secrete high levels of IL-6, IL-1β, and TNF-α (summarized in **Table 1**). Increased IL-6 expression is a hallmark of COVID-19 in association with acute respiratory distress syndrome (ARDS) and respiratory failure (35).

There is a higher risk of severe COVID-19 in patients with CVD, hypertension, and diabetes mellitus (35). Macrophages from diabetics were found to hyperpolarize to a pro-inflammatory phenotype *in vitro* when exposed to LPS and IFN-γ or TNF (36). M1 macrophage expression of TNF-α and IL-1β drive to renin angiotensin system (RAS)-dependent hypertension through type 1 receptor engagement on macrophages. Angiotensin II (AngII) contributes to hemodynamic injury and monocyte recruitment to the heart, vasculature, and kidneys where differentiation into M1 macrophages occurs (37). In COVID-19, ACE2 receptor engagement by viral spike protein reduces AngII degradation by ACE2, compounding AngII effects on hypertension (38). Atherosclerotic cardiovascular disease (ASCVD) is driven by chronic inflammation and is highly associated M1 macrophage expression of IL-6 and IL-1β. This inflammation is aggravated by risk factors such as smoking, obesity, and chronic viral infection. Macrophage infiltration into adipose tissue releases TNF-α resulting in serine phosphorylation of insulin receptor substrate 1 (IRS-1) which causes insulin resistance through impaired insulin signaling (39). M1 macrophages, the dominant macrophage subtype in ASCVD, stimulated with LPS demonstrate decreased ATP due to impaired oxidative phosphorylation, increased uptake of glucose, ROS production, inflammatory cytokine production, and lipid accumulation which accelerate disease progression in ASCVD. However, M2 macrophages are associated with atherosclerosis regression due to increased fatty acid oxidation and oxidative phosphorylation resulting in increased ATP (40).

Presence of FABP4+ alveolar macrophages and Ficolin1+ monocyte-derived macrophages (MDM)s in lungs of ARDS patients is associated with inflammation (35). FABP4+ macrophages are more dominant in milder disease whereas FCN1+ MDMs become the most dominant in ARDS patients. Lung macrophage populations are dysregulated as COVID-19 progresses in severity, leading to higher levels of macrophages and lower levels of T and natural killer cells in the lungs. This evidence combined with increased macrophage infiltration to lung in autopsy and a murine model indicates that monocyte recruitment to the lung fuels inflammation in severe COVID-19. FCN1+ macrophages have elevated expression of CCL2, CCL5, IL-8, CXCL9, CXCL10, and CXCCL11 which are products of IFN stimulated genes and suggest that FCN1+ macrophages contribute to hyper-inflammation (41). These cytokines are found to be upregulated in COVID-19 patients along with TGF-β and ISG, ITAM, and TRAM involved in cytokine storm (42). Near abolishment of FABP4+ macrophages in sever COVID-19 also contributes to dysregulated lung function (41).

Autopsy samples revealed macrophages enriched in tissue repair genes which is associated with fibrosis and suggests that macrophages contribute to fibrotic complications observed in COVID-19 patients on mechanical ventilation. It was posited that presence of viral protein in macrophages may be due to uptake of infected cells, and ACE-2 expression was not identified on most macrophages examined. It was suggested that inflammation may be responsible for triggering ACE-2 expression on macrophages. Macrophages containing virus were found to express IL-6 which is associated with lymphopenia. CD68+ macrophages containing viral nucleoprotein were found in kidneys of infected patients and are associated with kidney tubular damage. Macrophage accumulation is linked to damage in the kidneys, lungs, heart, liver, and muscles (42).

In a study of 54 COVID-19 patients, 28 of which had severe infection, all severe patients displayed macrophage activation syndrome (MAS) or low human leukocyte antigen (HLA-DR) expression along with lymphopenia (43). Lymphopenia is a common symptom in COVID-19 leading to reduced regulatory T cells (T_reg_) which is associated with dysregulated innate and inflammatory immune responses (35).

Interestingly, more than 1 in 10 patients who have cleared SARS-CoV-2 infection still suffer from persistent complications. This phenomenon has been termed long COVID. Up to 87.4% of acute COVID-19 patients reported at least one symptom persisting after several months. Females (23%) are more likely to develop long COVID than males (19%), and the condition is most common in middle aged individuals between 35 and 49 years of age. In children aged 2 to 11 years, it has been reported that 9.8% develop long COVID. Speculation on the source of long COVID suggests a multisystem disorder, prolonged effects of viral fragments, or autoimmune disease, but the actual cause has not yet been determined (44).

Of the current approved and investigated therapies for COVID-19, there are a mix of virus-specific and immune dysregulation targets (**Table 2**). Therapies such as baricitinib and dimethyl fumarate have direct acting effects on inflammation through the Jak STAT pathway (**Figure 1**), while inflammation mediated through the Mitogen-activated protein kinase (MAPK) pathway is targeted through downstream effects of therapies Tocilizumab (an IL-6 receptor inhibitor) and Dexamethasone (a MAPK phosphatase) (45, 46). Antibody-based therapy is a well utilized tool in the treatment of COVID-19. Intravenous immunoglobulin (IVIG) infusion therapy *via* convalescent plasma has shown efficacy in reducing mortality and disease severity in COVID-19 patients (47–53). The IgG in this therapy is isolated from the sera of convalescent patients with COVID-19 and is composed mostly of IgG1 and IgG2 (54, 55). IVIG therapy has been reported to prevent condition deterioration, recover lymphocyte counts, and prevent the need for mechanical ventilation and supportive care (56). IVIG therapy was first used as a therapeutic tool by Francesco Cenci for measles and was reported as a widely used treatment for the Spanish Influenza A (H1N1) pandemic of 1918 (57). IVIG has been reported as a safe treatment for other diseases including: Guillain-Barre syndrome, thrombotic thrombocytopenia, Goodpasture syndrome, and myeloma (56). There have been no associated adverse events for IVIG therapy and a randomized controlled trial has been initiated to evaluate high-dose IVIG in the management of severe COVID-19 (NCT04261426) (47, 58). IVIG is an immunomodulator of inflammation dysregulation which works to suppress the hyperactive immune response (cytokine storm) elicited by COVID-19, and as such timing of treatment initiation is key (56, 59). One study found that early administration of IVIG (within 3 days of hospital admission) resulted in a significantly shorter hospital stay (median 7 days) while late administration (greater than 7 days post admission) resulted in a significantly longer hospital stay (median 33 days) (60). Administering IVIG within 48 hours of ICU admission reduced the need for mechanical ventilation and boosted the immune response in newly infected patients (58, 61, 62). Another study of 26 patients admitted 10 days post disease onset found that administration of IVIG (5-day course) at an average of 13.2 days post disease onset increased lymphocytes and decreased inflammatory cytokines while reducing the 28-day mortality (IVIG treatment 4%; control 28%) (63). IVIG therapy has a limited EUA for high-titer dosing only and is recommended for early disease course treatment (64). Nonetheless, use of convalescent plasma has not conferred widespread use, as variable results and data demonstrating ineffective results have been reported in additional trials (65). Further, use of commercially produced monoclonal antibodies (summarized in **Table 1**) provides an efficacious modality to neutralize early infection in COVID-19 patients, and has become the standardized immunotherapy for first line treatment for mild COVID-19 therapies. Anti-spike protein monoclonal antibodies (Casirivimab, Imdevimab, Sotrovimab, Bamlanivimab, and Etesevimab) aim to block engagement of the ACE2 receptor and thus viral entry, while Remdesivir is a nucleoside analogue that targets viral production as a direct acting antiviral agent. The space for immunomodulatory therapies that target immune dysregulation and inflammation is clearly vital and should be further investigated in the context of viral infections.

**Table 1:** Summary of pathogenic features of viral species, associated cytokine profile, and drug targets discussed herein.

Virus	Macrophage Pathogenesis	Cytokine Upregulation									Drug Targets and Investigated Therapies
HIV	HAND, CVD	TNF-a, IL-1b, IL-6									
SARS-CoV-2	ARDS, ASCVD, DIC, lymphopenia, encephalopathy, stroke, CNS infection/demyelination, Guillain-Barre syndrome, acute fulminant cerebral edema	IL-6, IL-1β, TNF-a, CCL2, CCL5, IL-8, CXCL9, CXCL10, CXCCL1, TGF-β, ISG, ITAM, TRAM									
CHIKV	Joint pain	IL-12, IFN-a/β, IL-6, IFN-g, CCL2									
DENV	Hemorrhagic fever, shock syndrome	TNF-a, IFN-a, IL-1β, IL-8, IL-12, MIP-1a, RANTES									Jak-STAT Pathway – *Baricitinib, Ruxolitinib, Tofacitinib*
CMV	Microcephaly, mental/motor retardation, epilepsy, progressive vision and auditory deficits	TNF-a, IL-1β, IL-6									
HSV	Encephalitis, meningitis, cerebral palsy, cognitive retardation	CXCL10, TNF-a, CCL5, IL-1β, IL-6									BCL-2 Expression –*Venetoclax*
ZIKV	Microcephalus, cerebral atrophy, intracranial calcifications, hydrocephalus, encephalitis	IL-6, TNF-a, MCP-1, IP-10, IL-8								
IAV	ARDS, pneumonia		CXCL10	RANTES	TNF-a	MIP-1a	CCL2	IL-6	IL-8	TGF-β3	Nrf2 Pathway – *Dimethyl Fumurate*
		H5N1	**✓**	**✓**	**✓**	**✕**	**✓**	**✓**	**✓**	**✓**
		H9N2	**✓**	**✓**	**✓**	**✓**	**✓**	**✕**	**✕**	**✕**
		H7N9	**✕**	**✕**	**✓**	**✕**	**✕**	**✓**	**✓**	**✕**
		H1N1	**✓**	**✓**	**✓**	**✓**	**✕**	**✕**	**✓**	**✓**

TNF-α, IL-1β, and IL-6 are common pro-pathogenic, pro-inflammatory cytokines upregulated during infection. Upregulation of pro-inflammatory cytokines contribute heavily to viral pathogenesis and leads to development of co-morbidities in chronic infections.

**Table 2:** Current investigated and approved therapies for COVID-19.

COVID-19 Therapy	Original Indication	Mechanism of Action	Route of Administration	Disease State Approved For	Approval Status
Baricitinib	Rheumatoid arthritis	Jak ½ selective inhibitor	Oral*	Severe	EUA
Dexamethasone Sodium Phosphate	Endocrine, rheumatic, collagen, dermatologic, allergic, ophthalmic, gastrointestinal, respiratory, hematologic, neoplastic, edematous disorders/diseases	Adrenocortical steroid, immune modulator	IV, IM	Severe	FDA Temporary Policy for Compounding of Certain Drugs for Hospitalized Patients by Pharmacy Compounders not Registered as Outsourcing Facilities During the COVID-19 Public Health Emergency
Remdesivir	SARS-CoV-2	Nucleoside analogue, delayed chain termination of viral RNA	IV	Severe	FDA Approved
Tocilizumab	Rheumatoid arthritis, giant cell arteritis, systemic sclerosis-associated interstitial lung disease, polyarticular juvenile idiopathic arthritis, cytokine release syndrome	Anti-IL-6 receptor mAb	IV	Severe	EUA
Dimethyl Fumurate	Multiple sclerosis	Nrf2 pathway activator	Oral	N/A	RECOVERY trial candidate
Casirivimab & Imdevimab	COVID-19	Anti-human IgG1 mAb, bind to non-overlapping epitopes of SARS-CoV-2 spike RBD, blocks binding to ACE2	IV, SI	Mild/Moderate	EUA
Sotrovimab	COVID-19	Anti-human IgG1 mAb, binds to conservative epitope of SARS-CoV-2 spike RBD, non-competitive with ACE2 binding	IV	Mild/Moderate	EUA
Bamlanivimab & Etesevimab	COVID-19	Anti-human IgG1 mAb, binds to different but overlapping epitopes of SARS-CoV-2 spike RBD, blocks attachment to ACE2	IV	Mild/Moderate	EUA
Convalescent Plasma	Measles	Confers passive immunity by infusing naturally produced antibodies against a pathogen	IV	Mild/Moderate	Limited EUA

Therapies are currently under investigation, approved by the Food and Drug Administration (FDA), or have an emergency use authorization (EUA) from the FDA. Administration routes include oral, intravenous (IV), intramuscular (IM), and subcutaneous injection (SI). *Therapy can be alternatively administered when oral is not an option.

N/A, not available.

### Influenza Virus

Disease severity in IAV is associated with increased pro-inflammatory cytokine production (66, 67). Strains differ in cytokine profiles (**Table 1**). Infection of MDMs and alteration of cytokine production is a driver of pathogenesis in IAV. H5N1 and H9N2 are highly pathogenic. H9N2 induces CXCL10, RANTES, TNF-α, MIP-1α, and CCL2 expression (68). H5N1 induces TNF-α, CCL2, RANTES, CXCL10, IL-6, IL-8, and TGF-β3 (68, 69). H7N9 induces heightened IL-6, TNF-α, and IL-8 expression (69). H1N1 induces elevated TNF-α, IL-8, TGF-β3, CXCL10, RANTES, and MIP-1α expression (68, 69).

In *in vivo* IAV infection, an early increase in IFN-α, TNF-α, IL-1β, and IL-6 was observed in murine lungs. Nasal lavage samples from infected patients revealed heightened IFN-α, TNF-α, IL-8, IL-6, MIP-1α/β, and MCP-1 expression. Infected human monocytes and rat and murine macrophages produce IFN-α, TNF-α, IL-1, IL-6, MIP-1α/β, and RANTES. Pro-inflammatory cytokine production during IAV infection peaks 2-3 days post-infection. Massive bronchial infiltration of inflammatory cells, dominated by neutrophils, was observed in infected swine 24 hours post-infection. A decrease in viral titer is correlated with a decrease in neutrophil counts (70). Neutrophils cause tissue injury and have been targeted by anti-MIP-2 IgG therapy which decreased neutrophil counts to 37% of control in a murine model (71). Increased IFN-α, CCL7, and CD16-CD14+ monocytes are associated with disease severity in children while monocyte dysregulation also occurs in the elderly with decreased IFN type 1 signaling. Fatal pneumonia is triggered by increased IL-6 and ROS through stimulation of TLR-4 dependent alveolar macrophages by oxidized phospholipids (67). Curcumin has been investigated as a therapy because it inhibits the NF-κβ pathway and has been shown to decrease pro-inflammatory cytokine production in IAV infection (66). Autopsy samples revealed increased TNF-α and COX2. In a murine model, combined antiviral and COX inhibitor therapy has been shown to decrease lung pathology, pro-inflammatory cytokines, and lymphopenia (72). COX2 expression is induced by IL-1β which opens up a broader upstream target. However, COX inhibitors have been shown to over-suppress the inflammatory response to the host’s detriment (73).

### Chikungunya Virus

Both Chikungunya Virus (CHIKV) and Ross River Virus (RRV), alphaviruses, infect macrophages and their viral RNA and proteins have been observed in macrophages 18 months post-infection (74). Macrophages have been identified as the main reservoir in late stage CHIKV infection (75). Residual viral RNA and proteins may contribute to apoptosis, fibrosis, tissue injury, and inflammation. IL-12 is upregulated in CHIKV infection at time of infection and remains elevated in chronic infection. IL-12 promotes natural killer cell and macrophage activation. Inflammation results in joint pain and arthritis-like pathology (74). In a cynomolgus macaque model, CHIKV was found to target lymphoid tissues, muscles, joints, the liver, and the CNS during acute infection and persist in lymphoid tissues, joints, muscles, and the liver in late-stage infection. In non-human primates, major macrophage infiltration was observed in the spleen, liver, and lymph nodes while minor infiltration occurred in joints and muscles. Flow cytometry analysis revealed that 2/3 of CSF macrophages were activated in CHIKV infection. Within 12 days of infection, cytokine expression (**Table 1**) progressed from continuous macrophage activation (IFN-α/β, CCL2, IFN-γ), to pro-inflammatory (IFN-α/β, IL-6, IFN-γ), and finally to infiltration (CCL2). Peak viremia and pro-inflammatory cytokine expression were found to correlate with reduction in CD14+ cells (75).

### Dengue Virus

Dengue Virus (DENV) primary targets Kupffer cells and pulmonary macrophages while infected blood monocytes may disseminate DENV to tissues (76, 77). Macrophages and monocytes infected *in vitro* secreted TNF-α, IFN-α, IL-1β, IL-8, IL-12, MIP-1α, and RANTES (**Table 1**) (77). In post-mortem samples from 2 infants with primary DENV infection, it was found that DENV antigen was localized to Kupffer cells and splenic, thymic, and pulmonary macrophages. Another autopsy sourced study revealed DENV to be consistently associated with mononuclear phagocytes. Production of monokines or lymphokines resulting from T cell and infected macrophage interactions has been proposed as a source for rash, shock, and hemorrhage in DENV infection. Dengue hemorrhagic fever (DHF)/Dengue shock syndrome (DSS) is associated with DENV antibody being acquired pre-infection (76).

### Infantile Effects of Cytomegalovirus, Herpes Simplex Virus, and Zika Virus

*In vivo* murine microglia with a pro-inflammatory phenotype had elevated IL-6, IL-1β, and TNF-α expression that correlated with increased toxicity to fetal neuronal progenitor cells (NPCs) (**Table 1**) (78). Hofbauer cells, a target of Zika Virus (ZIKV) have access to fetal blood vessels which has been proposed as a route of viral dissemination to the brain. The main target of ZIKV are NPCs in the brain. Decreased cortical neurons and ultimately a smaller cerebral cortex (microcephaly) result from NPC reduction (79). Induced pluripotent stem cell-derived microglia-like cells have the ability to propagate ZIKV *in vitro* which supports their role as a microglial reservoir in ZIKV. Several cytokines are upregulated during ZIKV infection (IFN-α, IL-6, MCP-1, IP-10, and IL-8) (**Table 1**), but flaviviruses have been shown to evade the innate immune response by blocking Jak STAT pathway mediated IFN production. Treatment with known Jak inhibitor ruxolitinib elucidated the role of Jak STAT in cell permissiveness to ZIKV infection and as a regulator of mature virion production (80–82). It is important to understand these data in the context of time of addition and that these are *in vitro* studies that cannot model the impact of Jak ½ inhibition on disease pathogenesis and spreading.

Cytomegalovirus (CMV) can cross the placenta and infect the fetal brain. CMV infection in the first two trimesters in severe due to interference with placental development. CMV targets microglia, brain macrophages (CAM), and NPCs. An increase in microglia and macrophages due to recruitment within the brain and migration from the meninges was observed in murine CMV infected brains. CMV infected microglia secrete TNF-α, IL-1β, and IL-6 while infected fetal astrocytes secrete CCL2 to recruit microglia. Fetal astrocytes and neurons are infected by Herpes Simplex Virus (HSV) but microglia do not support viral replication. Myeloid dysregulation is actually protective in HSV because infected microglia secrete CXCL10, TNF-α, CCL5, or IL-1β. CXCL10 reduces viral replication in neurons and elevated IL-6 protects against neuronal loss (78).

## Neurological Complications of Macrophage-Driven Pro-Viral Events

### Myeloid Driven Comorbidity in HIV-1

HIV-1-associated neurocognitive disorder (HAND) and cardiovascular disease (CVD) are two comorbidities that result from persistent basal inflammation associated with the HIV-1 reservoir and chronic immune activation despite ART (83). In cerebrospinal fluid (CSF) from virologically suppressed individuals, gene expression in the myeloid subset was found to overlap that of neurodegenerative disease-associated microglia (84). In another study of 69 virologically suppressed individuals where ART was started during chronic infection, 33 had detectable HIV-1 DNA in the CSF. This supports that the CNS is a site of viral persistence despite ART suppression. The same study also found a significant association between neurocognitive dysfunction and detectable CSF associated HIV-1 DNA (85). In infants, HIV-1 disease progression is significantly faster compared to adults. Before ART was common practice, half of infantile infections resulted in progressive HIV-1-1 encephalopathy, targeting microglia, leading to microcephaly and developmental delays. Chronic neurological impairment is still observed under ART. Infected microglia release pro-inflammatory cytokines and reactive oxygen species (ROS) that contribute to neurotoxicity and neural injury (78).

Presence of infected and activated mononuclear phagocytes has been consistently linked to neuronal injury. Mononuclear phagocytes produce pro-inflammatory cytokines and neurotoxic products such as TNFα, IL-1β, IL-6, nitric oxide (NO), and glutamate among others (**Table 1**). Cathepsin B secretion from MDMs is elevated in response to HIV-1-1 infection. HIV-1 infected macrophage secreted Cathepsin B is associated with neuronal apoptosis (86). Microglia are the main target of HIV-1 infection in the brain. Infected microglia upregulate glutaminase C, an enzyme responsible for generating glutamate in the CNS, which has been confirmed in post-mortem tissues. HIV-1 infected macrophages have also been suggested to increase glutamate levels through upregulation of glutaminase (87, 88). Glutamate is neurotoxic in high concentration. Glutamate levels in the CSF have been directly correlated with severity of dementia and degree of brain atrophy (89). Glutaminase localizes to the inner mitochondrial membrane and catalyzes deamination of glutamine, thus producing glutamate. HIV-1 infected microglia mediate neurotoxicity through the N-methyl-D-aspartate (NMDA) receptor, for which glutamate engagement has been identified as a critical component of HAND excitotoxicity (89).

### Myeloid Driven Complications in COVID-19

About 36% of acute COVID-19 patients develop neurological symptoms regardless of pre-existing neurological disorders. Inflammation-induced dissemination intravascular coagulation (DIC) is a potential outcome of COVID-19 and can cause cerebrovascular ischemia leading to ischemic stroke. At time of discharge, one third of COVID-19 patients were found to have evidence of cognitive and motor impairment. Systemic inflammation is associated with cognitive decline and neurodegenerative disease leading to speculation in the literature that COVID-19 survivors may experience future neurodegeneration. ARDs, a common development in severe COVID-19, is also associated with cognitive decline and executive dysfunction (90). IL-6 is upregulated in patients with neurological symptoms in acute phase of inflammation (91). In a study of previously healthy children with multisystem inflammatory syndrome, 12% developed life-threatening conditions clinically associated with COVID-19 including severe encephalopathy, stroke, CNS infection/demyelination, Guillain-Barre syndrome, and acute fulminant cerebral edema. Of 43 patients who developed severe COVID-19 neurologic involvement, 40% had accrued new neurological deficits at time of discharge while 26% died (92). In COVID-19 patients with neurologic involvement, 50% were found to be hospitalized compared to 19% of non-neurologic COVID-19 (91).

ApoE4 is associated with heightened risk for development of severe COVID-19. ApoE4 is a genetic risk factor for Alzheimer’s disease and neuroinflammation. Neuronal extracellular vesicles (nEVs) have been found to increase inflammation and neurodegenerative proteins in COVID-19 patients. It is unknown if these proteins are transient. It has been suggested that transient neurodegenerative proteins would reflect ongoing neuroinflammation and elimination of toxic proteins for neurons, whereas long-term may indicate chronic neuroinflammation or developing neurodegeneration. nEVs promote neurogenesis, normal signaling in the CNS, and can remove damaged proteins but also spread toxic amyloid beta peptide (Aβ) and tau, a microtubule associated protein, between cells (91). Aβ accumulation in the brain has been proposed to be an early event in Alzheimer’s disease pathogenesis (93). Tau is a main component in intracellular filamentous inclusions in tauopathies like Alzheimer’s disease (94).

### Effects of Pre- and Post-Natal Infections

Microcephalus cerebral atrophy, intracranial calcifications, hydrocephalus, and encephalitis are potential outcomes of ZIKV and CMV fetal infection while HSV and HIV-1 fetal infection can lead to encephalitis, meningitis, cerebral palsy, and cognitive retardation. CNS macrophages develop early on and activation of these macrophages and inflammation can negatively affect the developing fetal brain. CMV fetal infection has been linked to microcephaly, mental and motor retardation, epilepsy, and progressive vision and auditory deficits (78). Several cases of autism resulting from CMV infection have been documented (95, 96). Elevated pro-inflammatory cytokine levels from pre-natal infection in the mother can compromise placenta barrier integrity and induce heightened cytokine production in the fetus. IL-6, IL-1β, and TNF-α can interfere with neuronal network development. Elevated IL-8 or TNF-α in the mother has been correlated with schizophrenia in the fetus. HSV infection in the CNS can result in herpes simplex encephalitis (HSE) and aseptic meningitis and can lead to neurological abnormalities in more than 50% of patients despite treatment. Macrophage dysregulation in HSV infection mitigates neurological deficits and is therefore protective (78).

## Targeting Macrophage-Driven Pro-Viral Events

### Pro-Viral Events

GM-CSF drives macrophage differentiation *via* activation of the Jak STAT pathway *via* JAK2 autophosphorylation (97). MDMs are activated and recruited by activated natural killer and T cells *via* GM-CSF, TNF, and IFN-γ. In COVID-19, accumulation of oxidized phospholipids in the lung activates MDMs through the TLR4-TRAF6-NF-κβ pathway (42). Jak 1 (IL-2, IL-7, IL-15, IL-6, IFN-α/β) and Jak 2 (IL-6, GM-CSF, IFN-γ) are activated by pro-inflammatory cytokines and in turn phosphorylate STAT 1, 3, and 5 which are then translocated into the nucleus where they bind to the promoter region of pro-inflammatory genes and promote transcription (98–101). Jak STAT activation in HIV-1 results in reduction of CD4+ cell counts, decreased IL-7R expression indicating elevated receptor engagement and ultimately increased homeostatic proliferation, and increased PD1 and CD38 expression indicating immune activation (99).

A downstream effect of Jak STAT activation is BCL-2 expression. BCL-2 is a pro-survival marker that contributes to the longevity of the HIV-1 reservoir. The reservoir is a major source of low-level inflammation despite ART. BCL-2 is upregulated in HIV-1 infection due to an increase in phosphorylated STAT5 that promotes transcription of the BCL-2 gene. Within the BCL-2 protein family, there are pro-survival (BCL-2, Mcl-1, BCL-XL) and pro-apoptotic (Bak, Bim, Bad, Bax) proteins. Pro-survival proteins sequester host BH3 which binds to pro-apoptotic proteins under normal conditions, oligomerize in the mitochondrial outer membrane resulting in permeabilization and release of cell death factors including cytochrome C. Cytochrome C activates caspase-9 to initiate a caspase cascade resulting in apoptosis (102, 103).

The nuclear factor (erythroid-derived 2)-like 2 (Nrf2) pathway is involved in cellular response to oxidative stress [Tecfidera package insert] (104). Nrf2 has anti-inflammatory function that works through inhibition of RNA polymerase II recruitment onto pro-inflammatory genes during transcription. This has been directly demonstrated with IL-6 in a murine model (104, 105).

Macrophage dysregulation in the context of heightened inflammation and factors contributing to reservoir establishment and maintenance should be further explored in HIV-1 and other viral infections.

### Immunomodulators

Immunomodulators discussed here particularly target points within the inflammatory cascade leading to macrophage dysregulation, key to pathogenicity of viral diseases discussed herein.

Jak inhibitors have been previously explored in the space of inflammatory disease and have been applied to viral infections in the cases of HIV-1-1 and COVID-19 in recent years (3, 106). BCL-2 inhibitors were originally developed as an anti-cancer therapy and have recently been evaluated in the context of HIV-1-1 (107). Using Food and Drug Administration (FDA)-approved compounds can potentially reduce the time of clinical translation due to a wealth of preliminary safety and efficacy profiling.

Tofacitinib is a Jak 3 selective inhibitor that is FDA approved for the indication of rheumatoid arthritis, however its utility in the space of HIV-1-1 in humans is diminished due to potential for Jak 3 mediated lymphopenia [Xeljanz package insert]. Ruxolitinib, a first generation Jak ½ selective inhibitor, has been evaluated *in vitro, ex vivo* and in humans for the indication of HIV-1-1 and has demonstrated a novel mechanism to block reservoir reseeding and maintenance (99, 108–110). The A5336 ACTG sponsored Phase 2a study demonstrated that ruxolitinib is safe and well tolerated in virally suppressed people living with HIV-1 and conferred a significant reduction in key markers of HIV-1-1 persistence including HLA-DR/CD38, sCD14, and reservoir lifespan marker Bcl-2. Baricitinib is a second generation Jak ½ inhibitor (FDA approved for rheumatoid arthritis and EUA for COVID-19) with an improved safety and efficacy profile *versus* ruxolitinib, including once per day (qd) dosing, renal clearance that mitigates many drug-drug interactions with hepatically cleared agents, and a markedly improved safety profile including FDA approval for chronic long-term use and approval in children as young as two years of age [Olumiant package insert]. Baricitinib was first evaluated in the viral infection space for HIV-1-1, and demonstrated a reversal in HIV-1 associated cognitive deficits along with a reversal of macrophage and microglial activation and key markers of CNS persistence in a murine model of HIV-1-1 in the CNS (111). Baricitinib was also recently evaluated under compassionate use for the indication of COVID-19 (106), and later found to reduce macrophage activation in a non-human primate model of SARS CoV-2 infection (112). Baricitinib later demonstrated a significant mortality benefit in humans in the NIH sponsored ACTT-2 trial (3), and the Lilly sponsored COV-BARRIER trial (Lancet RM, in press), which collectively conferred EUA approvals for baricitinib, and most recently as a monotherapy designation. To date, the class of Jak inhibitors, first crossed over to viral infections in the space of HIV-1-1, and now COVID-19, demonstrate a promising new class of agents; baricitinib in particular due to its safety and efficacy in humans in the COVID-19 space, coupled with its anti-HIV-1 properties, provides a unique opportunity for additional evaluation for HIV-1-1 cure studies, and possibly other viral infections with myeloid immune dysregulation.

Other agents have been explored within the repurposed space, which may impact myeloid immune dysregulation or, in the case of HIV-1-1, confer anti-HIV-1 effects that may down-regulate HIV-1-1 induced proliferation and expression ^77.^ Venetoclax is currently the only FDA-approved BCL-2 inhibitor and is indicated for acute myeloid leukemia, chronic lymphocytic leukemia, and small lymphocytic leukemia [Venclexta package insert] (113–115). Additional studies are needed with this class of agents to better understand if antiviral properties observed *in vitro* can crosstalk to relevant, safe, human studies for HIV-1-1 and other viral infections. Dimethyl fumarate is FDA-approved for relapsing multiple sclerosis and has been shown to activate the Nrf2 pathway *in vitro* and *in vivo* [Tecfidera package insert]. Nrf2 inhibits pro-inflammatory cytokine expression, disrupting the inflammatory cascade that exacerbates macrophage dysregulation (105). Dimethyl fumarate induces an antioxidant response and has been shown to suppress HIV-1 replication and macrophage-mediated neurotoxicity through attenuation of NF-κβ and monocyte recruitment to the CNS (116). Dimethyl fumarate has no black box warning and bid oral dosing of 120 mg for 7 days followed by 240 mg thereafter. The half-life of dimethyl fumarate is 1 hours and drug elimination is primarily through exhalation of carbon dioxide (CO_2_) with low level renal (16%) and fecal (1%) excretion. Dimethyl fumarate has not been approved in children as the safety and efficacy in this group is unknown [Tecfidera package insert].

Collectively, the field of immunomodulation for viral infections is a relatively new horizon, only recently explored through repurposed efforts, most notably through the Jak ½ inhibitor baricitinib, which has been applied for both HIV-1-1 and COVID-19 successfully. Recent insights across this potential application will provide additional data and understanding about the sentinel interplay between the immune system, macrophages, and treatment or prevention of viral infections.

## Conclusions

Macrophage dysregulation, particularly in the M1 subtype, leads to increased immune activation and inflammation that contributes to viral pathogenesis and development of comorbidities. Macrophage dysregulation is a pathogenic feature shared by multiple viruses and presents a broad target for antiviral treatments. In HIV-1, macrophages constitute a principal reservoir that is rarely affected by ART and which exists in tissues and the brain. Constant basal inflammation and immune activation lead to immune exhaustion and development of comorbidities such as CVD and HAND and can compound with other inflammatory diseases like diabetes. Macrophage contribution to viral pathogenesis extends beyond HIV-1 to SARS-CoV-2, Dengue, ZIKV, CMV, HSV, IAV, etc. A common feature of macrophage dysregulation is upregulation of pro-inflammatory cytokines such as IL-6, IL-1β, TNF-α, and IFN-α. Because this dysregulation transcends the BBB to reach the CNS, neurological symptoms are sometimes associated with macrophage dysregulation such as HAD and HAND in PWH and brain fog in people with long COVID. We must also consider this problem in the pre and postnatal scope. Macrophage dysregulation can cause physical and intellectual disabilities in infants. Downregulation of inflammation in viral infection should be further explored. Some immunomodulators such as ruxolitinib, baricitinib, venetoclax, and dimethyl fumarate have shown efficacy in this area. Furthermore, repurposing FDA approved compounds for this use represent an accelerated route from bench to clinic implementation. Inflammation in viral infections presents a broad target shared between multiple viruses. Focusing on this as a drug target, especially with repurposed immunomodulators, is an efficient and effective strategy in terms of cost, resources, availability, applicability, and impact on public health.

## Author Contributions

CG and RT conceptualized the review topic. MR performed the literature review, writing, and figure design. CS contributed to figure design. All authors revised and approved the submitted manuscript.

## Funding

Sources of funding for this work include the Dean’s Startup Package from Emory University’s School of Medicine and grant NIAID UM1AI164562-01 from the National Institutes of Health. These funders were responsible for compensating CG and MR’s roles in this work.

## Conflict of Interest

CG is the co-inventor of the internationally issued patent on Jak inhibitors for the treatment or prevention of HIV-1 and other viral infections; CG and Emory University receives royalties from Eli Lilly and Company for the sales of baricitinib for COVID-19.

The remaining authors declare that the research was conducted in the absence of any commercial or financial relationships that could be construed as a potential conflict of interest.

## Publisher’s Note

All claims expressed in this article are solely those of the authors and do not necessarily represent those of their affiliated organizations, or those of the publisher, the editors and the reviewers. Any product that may be evaluated in this article, or claim that may be made by its manufacturer, is not guaranteed or endorsed by the publisher.
